# Green-Mediated Synthesis of NiCo_2_O_4_ Nanostructures Using Radish White Peel Extract for the Sensitive and Selective Enzyme-Free Detection of Uric Acid

**DOI:** 10.3390/bios13080780

**Published:** 2023-08-01

**Authors:** Abdul Ghaffar Solangi, Aneela Tahira, Baradi Waryani, Abdul Sattar Chang, Tajnees Pirzada, Ayman Nafady, Elmuez A. Dawi, Lama M. A. Saleem, Mohsen Padervand, Abd Al Karim Haj Ismail, Kangle Lv, Brigitte Vigolo, Zafar Hussain Ibupoto

**Affiliations:** 1 Institute of Chemistry, Shah Abdul Latif University Khairpur Mirs, Khairpur Mirs 66111, Pakistan; ghaffarchemistry786@gmail.com (A.G.S.); aneela.tahira@salu.edu.pk (A.T.); tajnees@salu.edu.pk (T.P.); 2 Department of Fresh Water Biology and Fisheries, University of Sindh, Jamshoro 76080, Pakistan; baradiw@usindh.edu.pk; 3 Institute of Chemistry, University of Sindh, Jamshoro 76080, Pakistan; asattar.chang@usindh.edu.pk; 4 Department of Chemistry, College of Science, King Saud University, Riyadh 11451, Saudi Arabia; anafady@ksu.edu.sa; 5 Nonlinear Dynamics Research Centre (NDRC), Ajman University, Ajman P.O. Box 346, United Arab Emirates; e.dawi@ajman.ac.ae (E.A.D.); a.hajismail@ajman.ac.ae (A.A.K.H.I.); 6 Biomolecular Science, Earth and Life Science, Amsterdam University, 1081 HV Amsterdam, The Netherlands; lamysaleem@gmail.com; 7 Department of Chemistry, Faculty of Science, University of Maragheh, Maragheh P.O. Box. 55181-83111, Iran; padervand@maragheh.ac.ir; 8 College of Resource and Environment, South-Central Minzu University, Wuhan 430074, China; lvkangle@mail.scuec.edu.cn; 9 Institut Jean Lamour, Université de Lorraine, CNRS, IJL, F-54000 Nancy, France; brigitte.vigolo@univ-lorraine.fr

**Keywords:** non-enzymatic uric acid sensor, phytochemicals, radish white peel extract, biological matrix

## Abstract

The ability to measure uric acid (UA) non-enzymatically in human blood has been demonstrated through the use of a simple and efficient electrochemical method. A phytochemical extract from radish white peel extract improved the electrocatalytic performance of nickel–cobalt bimetallic oxide (NiCo_2_O_4_) during a hydrothermal process through abundant surface holes of oxides, an alteration of morphology, an excellent crystal quality, and increased Co(III) and Ni(II) chemical states. The surface structure, morphology, crystalline quality, and chemical composition were determined using a variety of analytical techniques, including powder X-ray diffraction (XRD), scanning electron microscopy (SEM), high-resolution transmission electron microscopy (HR-TEM), and X-ray photoelectron spectroscopy (XPS). The electrochemical characterization by CV revealed a linear range of UA from 0.1 mM to 8 mM, with a detection limit of 0.005 mM and a limit of quantification (LOQ) of 0.008 mM. A study of the sensitivity of NiCo_2_O_4_ nanostructures modified on the surface to UA detection with amperometry has revealed a linear range from 0.1 mM to 4 mM for detection. High stability, repeatability, and selectivity were associated with the enhanced electrochemical performance of non-enzymatic UA sensing. A significant contribution to the full outperforming sensing characterization can be attributed to the tailoring of surface properties of NiCo_2_O_4_ nanostructures. EIS analysis revealed a low charge-transfer resistance of 114,970 Ohms that offered NiCo_2_O_4_ nanostructures prepared with 5 mL of radish white peel extract, confirming an enhanced performance of the presented non-enzymatic UA sensor. As well as testing the practicality of the UA sensor, blood samples from human beings were also tested for UA. Due to its high sensitivity, stability, selectivity, repeatability, and simplicity, the developed non-enzymatic UA sensor is ideal for monitoring UA for a wide range of concentrations in biological matrixes.

## 1. Introduction

**Scheme 1:**
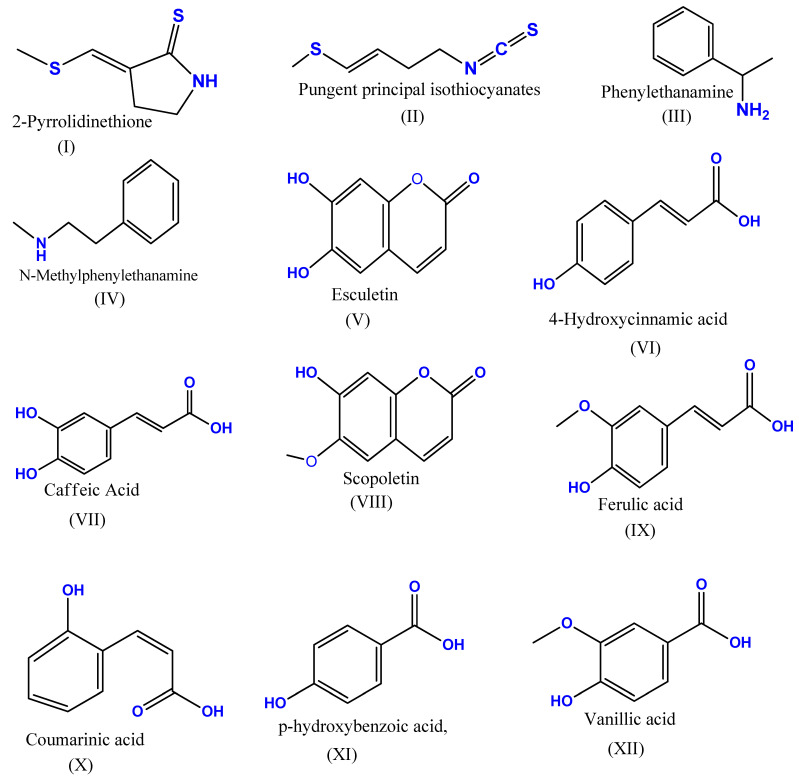
The various phytochemicals present in the radish (*Raphanus sativus*) peel extract.

A purine-based compound called alkaloids is used by our bodies to produce uric acid (UA) [1,2]. UA concentrations in serum samples range between 0.13 and 0.46 mM, whereas UA concentrations in urine samples range between 1.49 and 4.50 mM [3]. An abnormal level of UA in the human body is associated with several chronic and life-threatening diseases, including preeclampsia, arthritis, renal dysfunction, cardiovascular disease, obesity, high blood pressure, and kidney disease [4,5,6,7]. An increase in UA levels in blood serum has been identified as a major cause of cardiac disease [8]. For this reason, it is essential to measure and monitor UA in order to prevent the onset of dangerous conditions and to reduce the risk of premature death. Several analytical methods have been developed to measure UA as a result of this high importance, including colorimetric enzymatic tests [9]. Capillary electrophoresis method [10], surface-enhanced Raman scattering [11], liquid chromatography [12], electrochemical method [13], fluorescence spectroscopy [14], and chemiluminescence [15] are among the methods used. It is common for these analytical methods to be extremely expensive, time-consuming, and complex to operate. However, electrochemical methods are inexpensive, sensitive, and selective, thus they have been extensively studied [16,17,18,19,20,21]. A number of electrochemical approaches have been used to quantify UA’s high electrooxidation properties, including enzyme-based and enzyme-free methods [22,23,24,25,26]. Due to the high costs associated with enzyme immobilization and denatured issues with uricase enzyme during storage, non-enzymatic approaches have become increasingly popular as alternatives to enzyme-based approaches [27]. In non-enzymatic approaches, highly electrocatalytic materials are always desirable [16,18]. To realize non-enzymatic UA sensors quickly, it will be increasingly necessary to develop new materials with tailored surfaces that outperform electrocatalytic properties. This task, however, appears to be challenging because novel materials with tailored surfaces do not possess electrocatalytic properties. For the development of highly catalytic materials for non-enzymatic UA sensors, several challenges must be overcome, such as poor electrical conductivity, a limited number of catalytic sites, and chemical stability concerns. Therefore, numerous electrocatalytic materials have been developed and investigated for the development of non-enzymatic sensors [28,29,30,31]. A significant electrochemical activity of metal oxides makes them potentially suitable for this application [32,33,34]. As a result of its high electrical conductivity and favorable redox properties, a bimetallic oxide, particularly nickel–cobalt oxide (NiCo_2_O_4_), has been identified as a potential candidate for the development of non-enzymatic sensors. As NiCo_2_O_4_ nanostructures exhibit poor electrochemical performance due to their limited surface properties, they have been utilized in composites with other nanostructured materials, such as Fe_2_O_3_ @ NiCo_2_O_4_ [35], MnO_2_/NiCo_2_O_4_ [36], Co_3_O_4_/NiCo_2_O_4_ [37,38], and NiCo_2_O_4_ @ graphene [39]. It has been observed that composite NiCo_2_O_4_ systems exhibit enhanced charge transfer between electrode and analyte. Several morphologies of NiCo_2_O_4_ have been prepared, including nanotubes [37], nanorods [36], nanospheres [35], and nanosheets [33]. In spite of NiCo_2_O_4_’s diverse architecture, however, the material fails to perform as expected [35,36,38]. As a result, it is imperative to identify new strategies for improving the electrochemical performance of NiCo_2_O_4_ in order to develop more sensitive non-enzymatic UA sensors. Recent years have seen a great deal of interest in green chemistry due to its simplicity, eco-friendliness, low cost, and environmental friendliness [39,40,41,42]. Several natural products obtained from biomass wastes can be used to tailor the surface properties of nanostructured materials, including catalytic sites and charge transfer properties [41,42]. Only a few studies have been conducted on the synthesis of NiCo_2_O_4_ nanostructured materials from biomass wastes. The present study examines for the first time the effect of radish white peel extract (*Raphanus sativus*) on the morphology, crystal defect, and surface properties of NiCo_2_O_4_ that will be used for fabricating a non-enzymatic UA sensor. Radish white peel extract contains a wide range of phytochemicals, including alkaloids, glucosinolates, phenolic compounds, organic acids, anthocyanins, and isothiocyanates. [40,41] (Scheme 1).

The radish white peel extract possesses major phytochemicals, like as 4-hydroxycinnamic acid, caffeic acid, ferulic acid, and isothiocyanates, which were used as stabilizing, capping, and reducing agents, and played a significant role toward enhanced electrochemical properties of NiCo_2_O_4_ nanostructures.

In this study, we have used radish white peel extracts for the surface engineering of NiCo_2_O_4_ nanostructures by hydrothermal methods. The volume of extracts from peels was examined in order to determine the ideal concentration at which NiCo_2_O_4_ nanostructures could be scaled up for high performance production. An extensive linear range of UA detection was detected using surface-modified NiCo_2_O_4_ nanostructures ranging from 0.1 mM to 8 mM.

## 2. Experimental Section

### 2.1. Chemicals Used

In this study, cobalt chloride hexahydrate (CoCl_2_·6H_2_O), nickel chloride hexahydrate (NiCl_2_·6H_2_O), glucose, lactic acid, uric acid, sodium chloride, potassium chloride, ascorbic acid, sodium hydroxide, urea, hydrochloric acid, disodium phosphate, and monopotassium phosphate were applied without pretreatment. We obtained all analytical grade chemical reagents from Sigma Aldrich, Karachi, and Sindh Pakistan. UA detection was performed by preparing the desired solutions in deionized water, followed by preparing a buffer solution containing 0.1 M phosphate at pH 7.0 for UA detection.

### 2.2. Green-Mediated Synthesis of NiCo_2_O_4_ Nanostructures Using Radish White Peel Extract

**Scheme 2:**
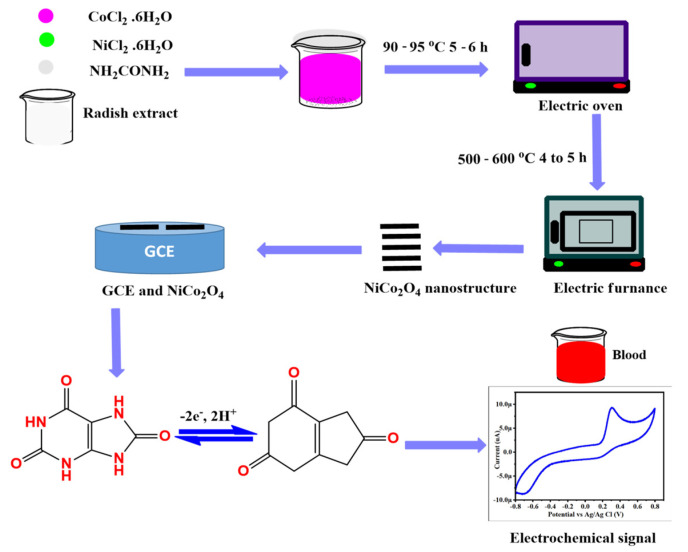
Stepwise synthesis of NiCo_2_O_4_ nanostructures using radish white peel extract during hydrothermal method, UA detection, and real blood sample analysis.

A hydrothermal method was used for phytochemical synthesis of NiCo_2_O_4_ nanostructures. The radish white was purchased, washed with deionized water, and allowed to dry at room temperature prior to the growth process. Small pieces of radish white were chipped after the peel had been removed. An automatic juicer was then used to collect juice from the peel. An extract of the peel was used in the subsequent synthesis of NiCo_2_O_4_ nanostructures. NiCo_2_O_4_ nanostructures were prepared using 0.1 M cobalt chloride hexahydrate, 0.1 M urea, and 0.015 M nickel chloride hexahydrate in 100 mL of deionized water through a hydrothermal process. Various amounts of radish white peel extract were used to synthesize NiCo_2_O_4_ nanostructures. The pH of the precursor solution was 8.2 and 7.1, respectively. Afterward, the growth solutions were sealed with aluminum sheets and grown at 95 °C for five hours. Filter paper was used to collect the bimetallic hydroxide phase, and deionized water was used to wash it several times. The material was then thermally roasted for five hours at 500 °C after drying for 12 h. With the same procedure, pristine NiCo_2_O_4_ nanostructures were obtained without adding radish white peel extract. An illustration of the synthesis of NiCo_2_O_4_ nanostructures as prepared can be found in Scheme 2.

### 2.3. Physical Investigations on the Surface-Modified NiCo_2_O_4_ Nanostructures

An assessment of the morphology of NiCo_2_O_4_ nanostructures was carried out by scanning electron microscopy (SEM: Auto Fine Coater: JEC-3000FC, No. JSM-IT 100, JEOL Japan Model, Tokyo, Japan; the coating performed at 20 mA current for 60 s) using an accelerating voltage of 10 kV. We assessed the crystal quality of radish white peel extract-assisted NiCo_2_O_4_ nanostructures using powder X-ray diffraction (XRD) at 45 kV and 45 mA using CuK radiation (λ = 1.5418 Å) as an X-ray source. The localized nanoscale structure was studied using high-resolution transmission electron microscopy (HRTEM) at a voltage of 200 kV. Utilizing energy dispersive spectroscopy, we were able to quantify the elemental mapping. The chemical composition of the surface was determined using X-ray photoelectron spectroscopy (XPS) in high vacuum. As a reference binding energy, we calibrated the XPS data using C1s at 284.6 eV and deconvolved the XPS features using a Shirley-type background and Voigt curves.

### 2.4. Non-Enzymatic Sensing of UA onto Surface-Modified NiCo_2_O_4_ Nanostructures

A variety of electrochemical methods have been used to characterize the non-enzymatic UA sensor, including cyclic voltammetry, amperometry, electrochemical impedance spectroscopy, and linear sweeping voltammetry. The electrochemical evaluation of NiCo_2_O_4_ nanostructures was conducted using a three-electrode setup. Three electrodes were used: silver–silver chloride (Ag/AgCl, 3.0 M KCl) as a reference electrode, platinum sheet as a counter electrode, and glassy carbon electrode (GCE) as a working electrode. Prior to modification of the GCE, it was polished with an alumina paste of (0.3 µM) and silicon paper and then cleaned with deionized water. The material ink was prepared by dispersing 10 mg of NiCo_2_O_4_ nanostructures in 2.5 m of deionized water and 0.5 mL of Nafion (5%) in 2.5 m of deionized water. We achieved homogeneous material ink after 15 min of an ultrasonic bath. NiCo_2_O_4_ nanostructures were applied to the surface of GCE using a drop-cast method. A 10 mM stock solution of UA was prepared in a 0.1 M phosphate buffer solution of pH 7.0. Prior to dissolving the phosphate buffer solution, UA was dissolved in propanol. The selectivity of the non-enzymatic UA sensor was determined with 0.1 mM interfering species, like urea, lactic acid, glucose, ascorbic acid, potassium ions, and sodium ions, in the presence of the same concentration of UA. The linear range of the UA sensor was determined by CV and chronoamperometry methods using various UA concentrations dissolved in 0.1 M phosphate buffer solution (PBS) at pH 7.0. Using this method, it was possible to estimate the low limit of detection of the non-enzymatic sensor [43].

## 3. Results and Discussion

### 3.1. Structural and Morphological Investigations of Radish White Peel Extract-Assisted Synthesis of NiCo_2_O_4_ Nanostructures

**Figure 1:**
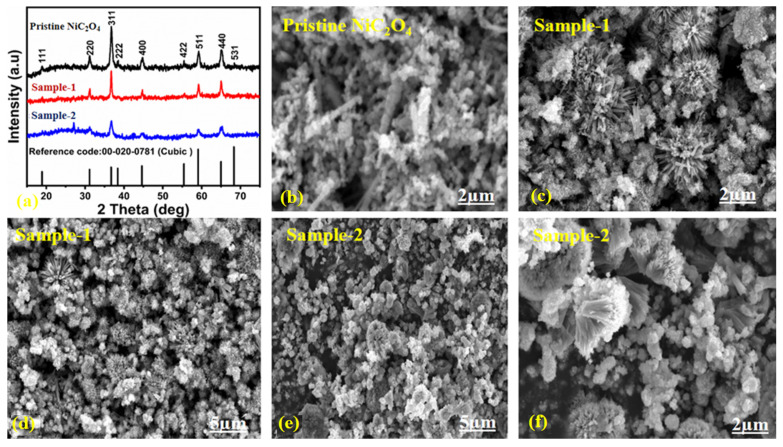
(**a**) Powder XRD diffraction patterns of pristine, 5 mL, and 10 mL of radish peel extract-assisted NiCo_2_O_4_ nanostructures, (**b**–**f**) Corresponding SEM images of (**b**) pristine, (**c**,**d**) 5 mL, and (**e**,**f**) 10 mL of radish peel extract-assisted NiCo_2_O_4_ nanostructures respectively.

**Figure 2:**
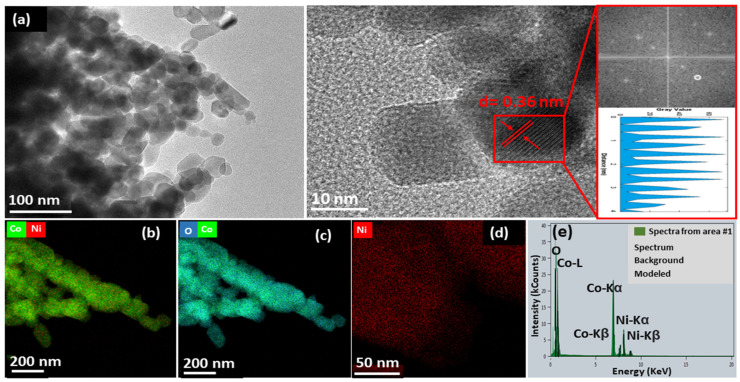
(**a**) TEM/HRTEM images of pristine NiCo_2_O_4_ nanostructures and FFT conversion at right side with d-spacing value; (**b**–**d**) corresponding elemental mapping of (**e**) EDS spectra of pristine NiCo_2_O_4_ nanostructures.

**Figure 3:**
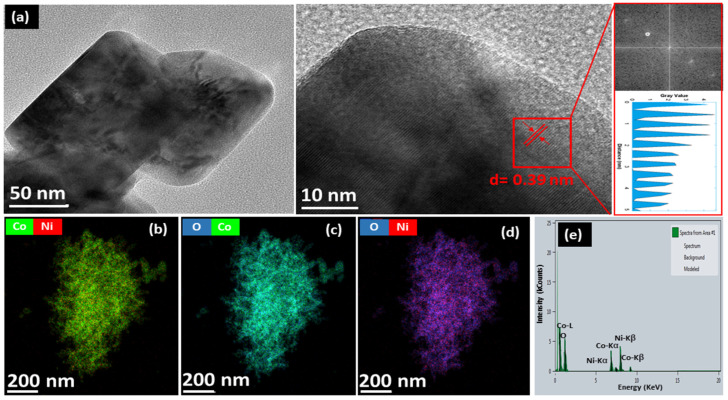
(**a**) TEM/HRTEM images of NiCo_2_O_4_ nanostructures prepared with 5 mL of radish white peel extract and FFT with d-spacing value on right-hand side; (**b**–**d**) elemental mapping of (**e**) EDS spectrum of NiCo_2_O_4_ nanostructures prepared with 5 mL of radish white peel extract.

In Figure 1a, we show diffraction patterns measured by powder XRD on NiCo_2_O_4_ nanostructures that reveal their crystal quality. Furthermore, we investigated the effects of phytochemicals present in the white peel extract of radish on the crystal quality of NiCo_2_O_4_ nanostructures. The reference card number 00-020-0781 confirms that the typical reflections of NiCo_2_O_4_ nanostructures are well matched with the cubic phase of NiCo_2_O_4_. The diffraction patterns of NiCo_2_O_4_ nanostructures have been identified as (111), (220), (311), (222), (400), (511), and (440), respectively, at 19.40, 31.30, 36.880, 38.590, 44.850, 59.410, and 65.300. The morphology of NiCo_2_O_4_ nanostructures prepared with radish peel extract was examined by SEM. This was performed to compare them with pristine NiCo_2_O_4_ nanostructures, as shown in Figure 1b–f. NiCo_2_O_4_ nanostructures with a high degree of heterogeneity exhibit a typical nanorod-like orientation as shown in Figure 1b. Figure 1c–f illustrates that radish white peel extract altered the morphology of NiCo_2_O_4_ nanostructures toward short-range nanoparticles, confirming the influence of phytochemicals on surface modification. As well as being much smaller in size, nanostructures have a size range between 50 and 100 nm, which is characteristic of nanoparticles. Figure 1f illustrates that radish white peel extract significantly affects the size and morphology of NiCo_2_O_4_ nanostructures. According to Figure 1f, these phytochemicals determined the morphology and allowed the formation of aggregated and irregularly oriented nanostructures with unfavorable surface properties when 10 mL of radish white peel extract was used during the growth process. A phytochemical from radish white peel extract, shown in Scheme 1, contains oxygenated groups that provide ample coordination with nickel and cobalt metallic ions during the growth process, which results in morphological changes from nanorods to nanoparticles. As-prepared NiCo_2_O_4_ nanostructures were examined with HRTEM images and Fourier transform (FFT) patterns in order to analyze the deep morphological features and to calculate atomic d-spacing. Figure 2a illustrates that the nanoparticles were assembled in order to form a nanorod-like structure. The HRTEM image shown in Figure 2a confirms the excellent crystallinity of NiCo_2_O_4_ nanostructures. According to Figure 2b, pristine NiCo_2_O_4_ nanostructures have a d-spacing of 0.39 nanometers. Using elemental mapping. Figure 2b–d illustrates a uniform distribution of Ni, Co, and O. Figure 2e illustrates the EDS spectrum, which shows Co, Ni, and O as the primary elements. A TEM and HRTEM examination of NiCo_2_O_4_ nanostructures, as shown in Figure 3a, was conducted along with the calculation of d-spacing. A TEM image revealed typical nanoparticle morphologies, and HRTEM confirmed the high crystal quality of NiCo_2_O_4_ nanostructures (Figure 3). Figure 3a shows that NiCo_2_O_4_ nanostructures exhibit a spinel crystal structure and a cubic phase as revealed by the FFT analysis. According to Figure 3b–d, the d-spacing value of this sample was 0.36 nm, in agreement with results published for NiCo_2_O_4_ nanostructures. An elemental analysis of these nanostructures utilizing 5 mL of radish white peel extract revealed a homogeneous distribution of Co, Ni, and O elements. As shown in Figure 3e, the EDS spectrum indicates that the surface-modified NiCo_2_O_4_ nanostructures prepared using 5 mL of radish white peel extract contain quantified amounts of Ni, Co, and O.

**Figure 4:**
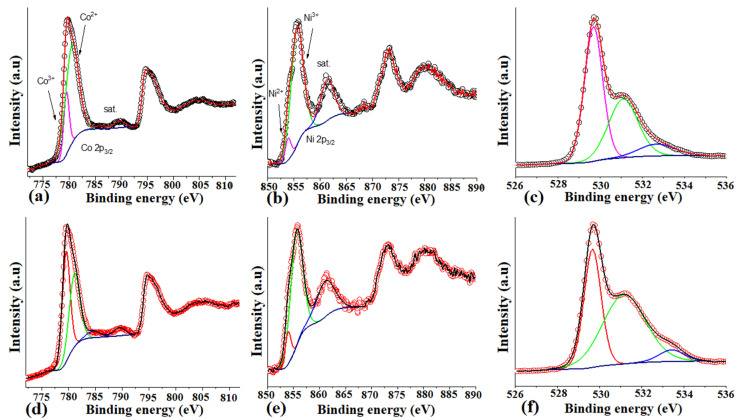
XPS-resolved Co 2p, Ni 2p, O1s spectra for pristine (**a**–**c**) and (**d**–**f**) XPS-resolved Co 2p, Ni 2p, O1s of 5 mL of radish peel extract-assisted NiCo_2_O_4_ nanostructures.

**Figure 5:**
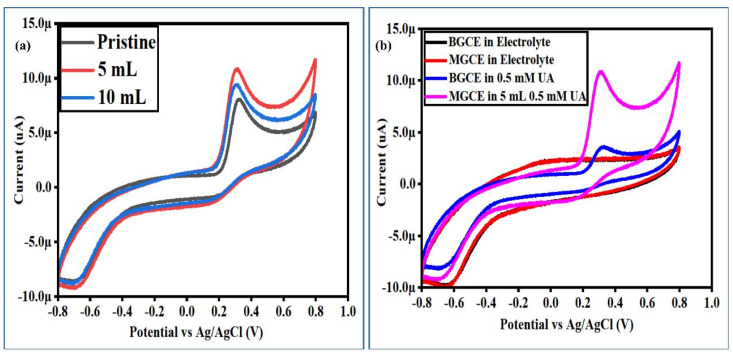
(**a**) Cyclic voltammograms at a scan rate of 50 mV/s of MGCE with 5 mL and 10 mL of radish white peel extract-assisted NiCo_2_O_4_ and pristine NiCo_2_O_4_-modified GCE in the presence of 0.5 mM of UA in 0.1 M PBS pH 7.0. (**b**) Cyclic voltammograms at 50 mV/s of bare GCE and modified with 5 mL of assisted NiCo_2_O_4_ in electrolyte and equal in the presence of 0.5 mM UA in 0.1 M PBS pH 7.0.

The presence of green organic recuing, capping, and stabilizing agents in the radish white peel extract have the capability to tailor the surface properties of nanostructured materials [28,42]. Using XPS analysis, we were able to determine the chemical states and surface species of NiCo_2_O_4_ nanostructures both in the absence and presence of radish white peel extract, as shown in Figure 4. The XPS study has demonstrated a significant role of a wide range of green organic reducing, capping, and stabilizing agents on the surface chemical composition of NiCo_2_O_4_ nanostructures. To fit the XPS binding energies of each element, a standard carbon binding energy was used. Using pristine NiCo_2_O_4_ nanostructures as an example, Figure 4a illustrates the Co 2p spectrum, which indicates that there are two types of Co chemical states. According to Figure 5a, the estimated peaks at 779.47 eV and 780.54 eV correspond to the oxidation states of Co^3+^ and Co^2+^, respectively. It is illustrated in Figure 5b how the Ni 2p spectrum can be fitted using Voigt’s method. The fitted data consist of two spin orbital doublet peaks, 853.88 eV and 855.60 eV, corresponding to the Ni^2+^ and Ni^3+^ chemical states. It should be noted that despite these limitations, the shakeup peaks have been identified, and the data of Ni 2p fitted to XPS agree reasonably well with the reported peaks [36,44]. Furthermore, Figure 4c displays the O 1s spectrum for pristine NiCo_2_O_4_ nanostructures in addition to the metallic chemical states. The material surface shows three peaks at 529.69 eV, 531.10 eV, and 532.68 eV, respectively, corresponding to metal–oxygen chemical bonds, oxygen ions, and physic/chemisorbed water. Previous studies [36,44] have detected different oxygenated species on the surface of pristine NiCo_2_O_4_ nanostructures. The spectrum of O^−1^ s at 529.69 eV and 531.10 eV was correlated with the O^2−^ species present on NiCo_2_O_4_ nanostructures. A similar XPS analysis was conducted on NiCo_2_O_4_ nanostructures prepared in the presence of 5 mL of radish white peel extract as shown in Figure 4d–f. A large peak was observed in the spectrum of Co 2p associated with oxidation states, such as Co^3+^ and Co^2+^, at 779.57 eV and 781.12V, respectively. By contrast, Ni^2+^ and Ni^3+^ were observed at 853.96 eV and 855.61 eV, respectively. Furthermore, three contributions were observed in the O1s spectrum at 529.67 eV, 531.16 eV, and 533.42 eV, corresponding to the metal–oxygen bonds, oxygen ions (O_2_), and physic/chemisorbed water on the surface of the material. The XPS study has revealed that green organic reducing, capping, and stabilizing agents from radish white peel extract have a relatively high concentration of oxygen ions (O^−^) and Co^3+^ compared to pristine NiCo_2_O_4_ nanostructures. These surface properties are highly desirable for a catalytic reaction [36,44]. Furthermore, the green organic reducing, capping, and stabilizing agents have induced a high abundance of Co^3+^/Co^2+^ and Ni^2+^/Ni^3+^ metallic positively charged ions in the surface of NiCo_2_O_4_ nanostructures. Consequently, these metallic ions have offered a high density of catalytic sites for the favorable electrocatalytic reaction of UA.

### 3.2. Non-Enzymatic Uric Acid (UA) Oxidation on the Surface-Modified NiCo_2_O_4_ Nanostructures with Radish White Peel Extract

**Figure 6:**
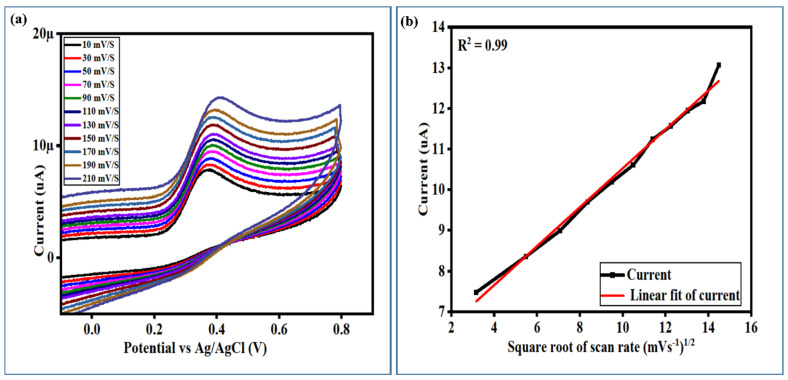
(**a**) Cyclic voltammograms at a scan rate of 50 mV/s of MGCE with 5 mL of radish white peel extract-assisted NiCo_2_O_4_-modified GCE in the presence of 0.5 mM of UA in 0.1 M PBS pH 7.0. (**b**) Linear plot of peak current against square root of scan rate.

NiCo_2_O_4_ nanostructures were characterized electrochemically using a three-electrode cell configuration in order to detect UA. Figure 5a illustrates preliminary studies conducted with cyclic voltammetry to identify the most efficient NiCo_2_O_4_ nanostructures for the oxidation of UA. A CV curve was measured at 50 mV/s using both pristine NiCo_2_O_4_ nanostructures and NiCo_2_O_4_ nanostructures, which had been surface-modified with 5 mL and 10 mL of radish white peel extract in the absence or presence of UA. A CV curve, such as that shown in Figure 6a, is representative of electrochemical catalytic signals obtained from pristine and surface-modified NiCo_2_O_4_ nanostructures placed in 0.5 mM UA in a phosphate buffer solution at pH 7.0. When comparing NiCo_2_O_4_ nanostructures prepared with 5 mL of radish white peel extract with pristine and surface-modified NiCo_2_O_4_ nanostructures soaked in 10 mL of radish white peel extract, it is evident that the electrocatalytic properties of these nanostructures are well described when compared to pristine and surface-modified NiCo_2_O_4_ nanostructures soaked in 10 mL of radish white peel extract. This is due to the enhanced conductivity of the material and the enriched surface sites on the surface. Due to the poor catalytic properties and the electrical conductivity of NiCo_2_O_4_, the CV curve shows that the peak current is relatively low when UA is oxidized on pristine NiCo_2_O_4_ nanostructures. However, the electrochemical performance of NiCo_2_O_4_ nanostructures was strongly dependent on the volume of radish white peel extract, as evidenced by the limited electrochemical signal in Figure 5a for a sample containing 10 mL of radish white peel extract. For the large-scale synthesis of NiCo_2_O_4_ nanostructures that are highly desirable for electrochemical applications, 5mL of radish white peel extract appears to provide the best conditions. Moreover, NiCo_2_O_4_ nanostructures prepared using 5 mL of radish white peel extract and bare glassy carbon electrodes were tested in the presence of 0.5 mM UA and only in the presence of a phosphate buffer solution at pH 7.0, as shown in Figure 6b. UA is believed to cause the signal to originate primarily from NiCo_2_O_4_ nanostructures; however, the bare glassy carbon electrode, as shown in Figure 5b, did not demonstrate any electrochemical signal for either the electrolyte or analyte. Based on the CV analysis, NiCo_2_O_4_ nanostructures synthesized with radish white peel extract were only effective for driving UA oxidation in the phosphate buffer solution, thus full non-enzymatic UA sensor characterization was carried out.

When UA is oxidized on NiCo_2_O_4_ nanostructures, electrons are transferred from UA to Co^3+^ and Ni^3+^ ions and reduced to Co^2+^ and Ni^2+^. In addition to altering the size and surface properties of NiCo_2_O_4_ nanostructures, phytochemicals in radish white peel extract enhanced the charge transfer between the electrode and analyte solution. Figure 6a shows the electrode kinetics at various scan rates using CV analysis in a solution containing 0.5 mM UA. It is evident from this example that increasing scan rates linearly increase peak current. This confirms the diffusion-controlled processing of the modified GCE with NiCo_2_O_4_. As shown in Figure 6b, a linear plot was obtained by plotting the peak current against the square root of the scan rate. The purpose of this was to simplify the understanding of electrode kinetics. NiCo_2_O_4_ nanostructure-based electrodes have been found to have a high scan rate in recent studies. According to CV analysis, UA oxidation is accompanied by two electron and proton transfers [44,45,46,47]. Also, the observed regression coefficient value (R^2^—0.99) supports the analytical aspects of an enzyme-free UA sensor for precise and accurate quantification of UA. Therefore, surface adsorption and electrochemistry were responsible for controlling the UA oxidation reaction.

**Figure 7:**
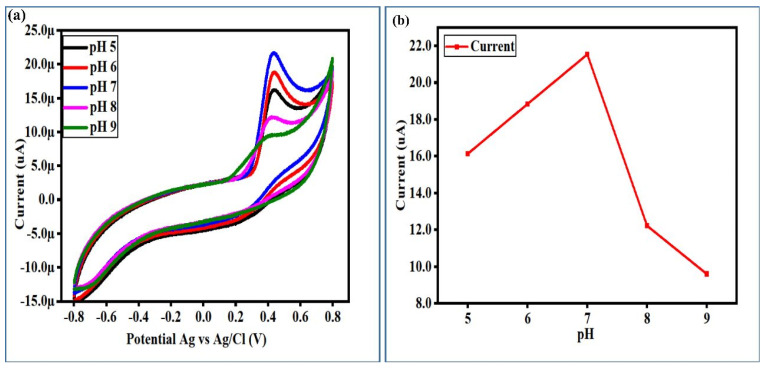
(**a**) Cyclic voltammograms at a scan rate of 50 mV/s of MGCE with 5 mL of radish white peel extract-assisted NiCo_2_O_4_-modified GCE in the presence of different pH values of 0.5 mM of UA in 0.1 M PBS. (**b**) Linear plot of peak current versus different pH values of 0.5 mM of UA in 0.1 M PBS.

Our study examined the effect of pH on the oxidation process of NiCo_2_O_4_ nanostructures prepared with 5 mL of radish white peel extract. As a result of the pH change of 0.5 mM of UA, CV curves for NiCo_2_O_4_ nanostructures are illustrated in Figure 7a. We evaluated UA sensor performance at pH 7.0 based on the shape and current of the peak, which were more apparent at this pH level. A pH study indicated that NiCo_2_O_4_ nanostructures are strongly regulated by the pH of the analyte solution when it comes to their activity. Such aspects have previously been examined in a study [48]. NiCo_2_O_4_ nanostructures are limited in their ability to function under different pH conditions of analyte solutions. Consequently, the catalytic sites are diminishing, the material is unstable, and the surfaces are etched [49]. As a result of this experiment, the pH of the UA solution was varied between 6.0, 7.0, 8.0, and 9.0. As a result, CV displayed a stable response at pH 7.0, and all electrochemical measurements were made at this pH level. To facilitate interpretation of CV curves, a line plot was also made of the effect of pH on the UA solution. Figure 7b illustrates that pH 7.0 resulted in the highest peak current, which improved charge transfer and increased the NiCo_2_O_4_ nanostructures’ electrochemical activity. UA oxidation occurs when two protons are lost and is suppressed by UA’s low pH of 7.0. However, at higher pH levels, NiCo_2_O_4_ nanostructures are likely to be etched, resulting in poor performance.

### 3.3. The Calibration Plots, Stability, Repeatability, and Selectivity Studies of a Newly Developed Non-Enzymatic UA Sensor Based on Surface-Modified NiCo_2_O_4_ Nanostructures

**Figure 8:**
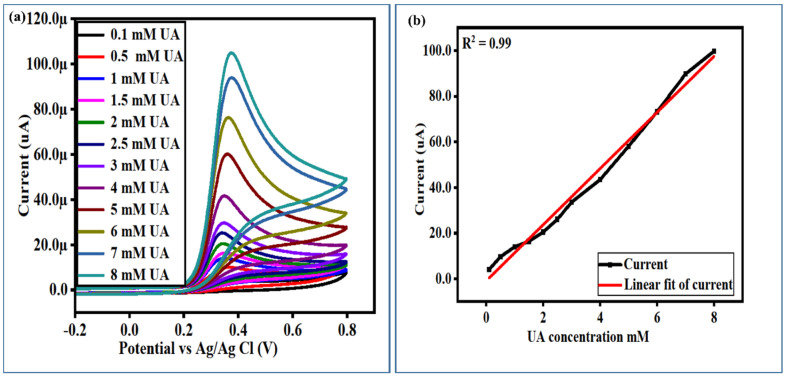
(**a**) Cyclic voltammograms at a scan rate of 50 mV/s of MGCE with 5 mL of radish white peel extract-assisted NiCo_2_O_4_ in the presence of various concentrations of UA in 0.1 M PBS pH 7.0. (**b**) Linear plot of peak current versus successive increase in UA concentrations.

**Figure 9:**
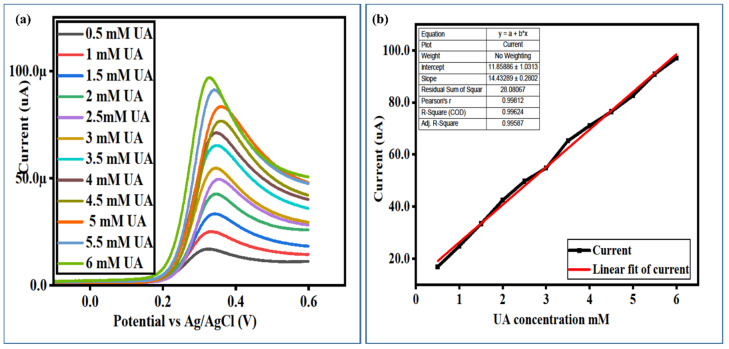
(**a**) Linear sweep voltammetry at a scan rate of 10 mV/s of MGCE with 5 mL of radish white peel extract-assisted NiCo_2_O_4_ in the presence of various concentrations of UA in 0.1 M PBS pH 7.0. (**b**) Linear plot of peak current versus successive increase in UA concentrations.

**Figure 10:**
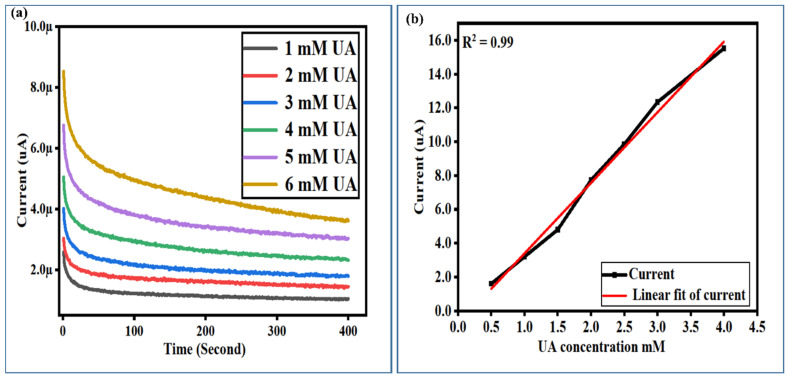
(**a**) Chronoamperometric response curves measured at an applied potential of 0.3 V of MGCE with 5 mL of radish white peel extract-assisted NiCo_2_O_4_ in the presence of various concentrations of UA in 0.1 M PBS pH 7.0. (**b**) Linear plot of peak current versus successive increase in UA concentrations.

Analyses of the linear range and limit of detection of UA were conducted using NiCo_2_O_4_ nanostructures prepared with 5 mL of radish white peel extract. The linear range of a non-enzymatic UA sensor was evaluated by varying electrochemical modes in order to maintain the sensing range of each electrochemical mode. The linear range of UA was first investigated by CV at 50 mV/s in a phosphate buffer solution at pH 7.0 at different concentrations of UA. The linear range of UA was determined to be between 0.1 mM and 8 mM, and the peak current for UA oxidation increased linearly with increasing UA concentration, as shown in Figure 8a. Based on this study, it can be concluded that UA non-enzymatic sensors have demonstrated a wide linear range to date [13,50,51,52,53,54,55,56] as well as the low detection limit of 0.005 mM, indicating a high degree of applicability for the monitoring of UA at either low or high concentrations in real samples. In addition, the linear plot shown in Figure 8b was calculated by selecting the oxidation peak current for each UA concentration against the UA concentration in order to determine the accuracy and precision of a newly developed non-enzymatic UA sensor. It appears that UA sensors have the ability to monitor a wide range of UA concentrations from practical samples based on the linear plot of the CV results. On the basis of the methods reported in the literature, a limit of detection (LOD) and a limit of quantification (LOQ) were estimated [56]. In this study, the LOD and LOQ were determined to be 0.005 mM and 0.008 mM, respectively. Table 1 compares the wide linear range of UA detection and the low LOD of the non-enzymatic UA sensors presented with the existing UA biosensors. Following the analysis of the comparisons, it became evident that the proposed method can be of great interest as an alternative method for the detection of UA in real samples, where a wide linear range and a low limit of detection are desired. Additionally, linear sweep voltammetry (LSV) mode was used to estimate the calibration of the newly developed non-enzymatic UA sensor, and Figure 9a illustrates the range of UA detection obtained. Figure 9a illustrates that the proposed UA sensor configuration is capable of detecting UA over a wide linear range from 0.1 mM to 7.0 mM and producing a significant amount of current. When UA concentration increases in LSV measurements, there is a higher current generated, which indicates that the newly developed UA sensor is highly sensitive. Besides the LSV measurements, we also made a linear plot of peak current and UA concentrations (Figure 9b). The linear fitting of LSV curves reveals that the proposed non-enzymatic UA sensor has excellent analytical performance, as indicated by its regression coefficient value of 0.99. Based on full CV curves and half CV curves from LSV, the present UA sensor is capable of detecting a wide linear range of UA with precision and accuracy. Additionally, the linear range for another highly sensitive electrochemical mode of amperometry at 0.3 V was used to determine the linear range of the UA sensor. Figure 10a illustrates this range for various UA concentrations detected. UA concentrations between 0.1 and 6 mM were highly sensitive to the amperometric signal. Figure 10b illustrates a linear plot of the amperometric signal for these different concentrations of UA.

**Figure 11:**
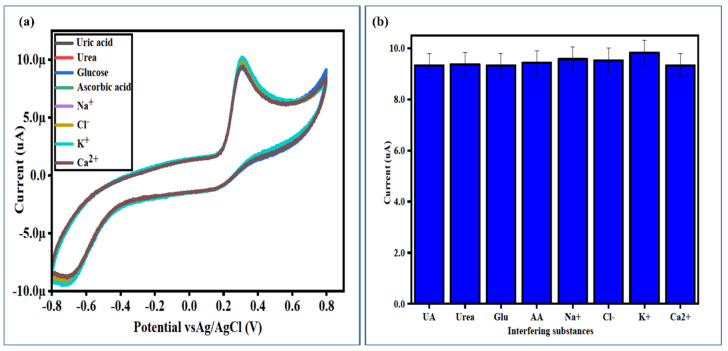
(**a**) Cyclic voltammograms at a scan rate of 50 mV/s of MGCE with 5 mL of radish white peel extract-assisted NiCo_2_O_4_ in the presence of 0.5 mM UA and other competing interfering agents in 0.1 M PBS pH 7.0; (**b**) bar graph of peak current with addition of interfering species for the illustration of the variation of the peak current.

A linear fitting of the UA detection resulted in excellent performance with strong analytical features. The results confirm the algorithm’s promising performance for the detection of UAs and its application in real-world sample analysis. Due to the high density of catalytic sites, enhanced electrical conductivity, and biomimetic compatibility of nanostructured material with the surface of GCE, UA detection on surface-modified NiCo_2_O_4_ nanostructures with radish white peel extract presented outstanding linearity and high sensitivity. The SEM, XRD, HRTEM, and XPS measurements demonstrated tailored morphology, excellent crystal quality, a significant amount of surface vacancies, and an abundance of Co(III) and Ni(II) chemical states, which led to the superior performance of NiCo_2_O_4_ nanostructures prepared with 5 mL of radish white peel extract. In order to evaluate the selectivity of a UA sensor before implementing it in actual sample analysis, we tested the proposed UA sensor in the presence of possible interfering species during detection of UA in real blood and urine samples. It was determined that the selectivity of the interfering compounds could be monitored by preparing NiCo_2_O_4_ nanostructures with radish white peel extract containing glucose, ascorbic acid, urea, lactic acid, mannose, sodium ions, chloride ions, potassium ions, and calcium ions, as shown in Figure 11. CV curves were measured following the sequential addition of interfering species to a solution of 0.5 mM UA. Based on Figure 11a, the interfering species had the same concentration as the UA. As a result of the sequential addition of interfering species in the presence of UA, UA was not affected in terms of oxidation peak position, drift in oxidation potential, and peak current, suggesting that the proposed UA sensor configuration has excellent selectivity and is suitable for the detection of UA in biological matrixes. In order to better visualize the variation in UA peak current after the addition of interfering species, a bar graph of the peak current was plotted. The change in peak current could be seen at less than 4%, as shown in Figure 11b. The excellent selectivity of the proposed UA sensor can be attributed to the surface modification of NiCo_2_O_4_ nanostructures with phytochemicals from radish white peel extract, which enabled it to detect specific electroactive molecules.

**Figure 12:**
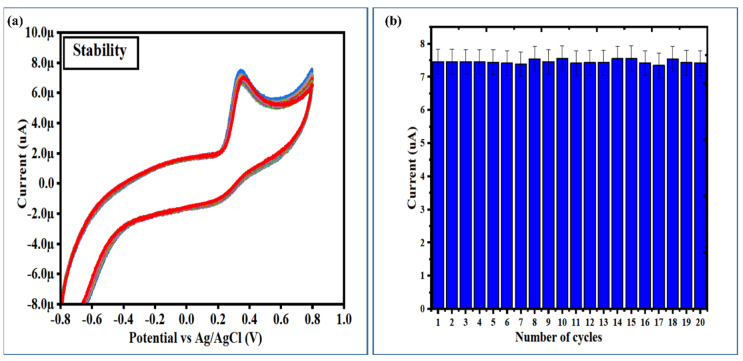
(**a**) Cyclic voltammograms at a scan rate of 50 mV/s of MGCE with 5 mL of radish white peel extract-assisted NiCo_2_O_4_ in the presence of 0.5 mM UA in 0.1 M PBS pH 7.0; (**b**) bar graph of peak current for the description of change in the peak current with increasing number of CV cycles. Linear plot of peak current versus successive increase in UA concentrations.

**Figure 13:**
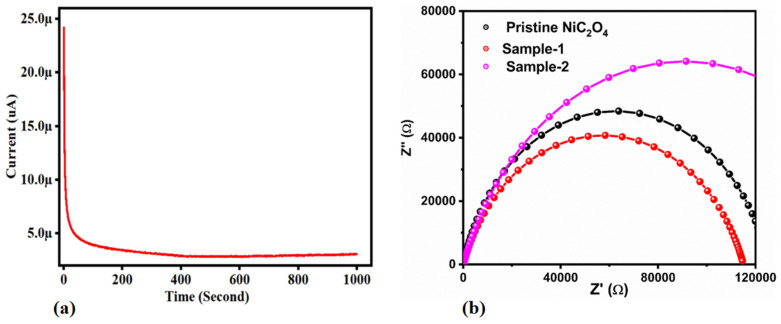
(**a**) Chronoamperometric response of MGCE with 5 mL of radish white peel extract-assisted NiCo_2_O_4_ in 0.5 mM prepared in 0.1 M PBS pH 7.0 for the demonstration of stability of modified electrode; (**b**) EIS Nyquist plots collected for the MGCE with pristine, 5 mL, and 10 mL of radish white peel extract-assisted NiCo_2_O_4_ in 0.5 mM UA using frequency range of 100 kHz to 1 Hz, amplitude of 10 mV, and biasing potential of 0.6 V.

We examined the repeatability of the modified UA sensor electrode by measuring 20 CV cycles at a scan rate of 50 mV/s in 0.5 mM. It was found that the device could be reused, as shown in Figure 12a. The long-term stability of UA biosensors is always a challenge, especially for enzymatic biosensors, thus we have designed a non-enzymatic UA sensor with potential application in real analysis. This is evident from the bar graph of peak current after several repeatable CV cycles as shown in Figure 12b. An error of less than 5% indicates the excellent analytical features of the method. A non-enzymatic UA based on surface-modified NiCo_2_O_4_ nanostructures can be used for long-term applications, since the material does not change surface features under ambient conditions. However, we examined the stability of NiCo_2_O_4_ nanostructures in 0.5 mM UA solution by measuring the amperometric response over a period of 1000 s. This is depicted in Figure 13a. According to the response time, the present UA sensor did not exhibit any current fluctuation for the selected time. Therefore, it is suitable for long-term applications.

Our results in terms of wide linear range, low limit of detection, excellent selectivity, stability, and repeatability confirmed the superior performance compared to other UA sensors/biosensors reported in the existing literature, as shown in Table 1. To support the electrochemical performance of the prepared NiCo_2_O_4_ nanostructures, electrochemical impedance spectroscopy (EIS) was performed using a sweeping frequency range of 100 kHz to 1 Hz, amplitude of 10 mV and biasing potential of 0.6 V. The Nyquist plots were measured for the three samples of NiCo_2_O_4_ nanostructures, including pristine sample 1 and sample 2 in 0.5 mM UA, and the EIS data were fitted with Z-view software as illustrated in Figure 13b. The intercept of the semicircle of Nyquist plots at high frequency represents electrolyte resistance. The arc of the Nyquist plots indicates the charge transport between the working electrode based on NiCo_2_O_4_ nanostructures and the 0.5 mM analyte solution [61]. The estimated values of charge-transfer Rct for pristine NiCo_2_O_4_ nanostructures, sample 1, and sample 2 in the presence of analyte were as 125,100 Ohm, 114,970 Ohm, and 181,270 Ohm, respectively. The EIS study has verified that sample 1 of NiCo_2_O_4_ nanostructures prepared with 5 mL of radish white peel extract has more favorable charge transport over the pristine and sample 2-based NiCo_2_O_4_ nanostructures; hence, we observed better electrochemical measurements of sample 1 toward the quantification of a sensitive UA signal.

### 3.4. Human Blood Sample Analytical Applications of Proposed Non-Enzymatic UA Sensor-Based NiCo_2_O_4_ Nanostructures

**Figure 14:**
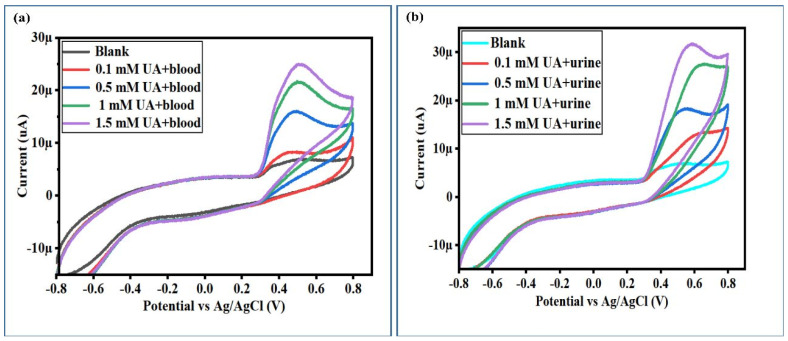
(**a**) Cyclic voltammograms at a scan rate of 50 mV/s of MGCE with 5 mL of radish white peel extract assisted NiCo_2_O_4_ for the quantitation of UA form diluted human blood real samples in 0.1M PBS pH 7.0 and successive addition method; (**b**) Cyclic voltammograms at a scan rate of 50 mV/s of MGCE with 5 mL of radish white peel extract assisted NiCo_2_O_4_ for the quantitation of UA form diluted human urine real samples in 0.1M PBS pH 7.0 and successive addition method.

The non-enzymatic UA sensor configuration presented in Figure 14a,b has been evaluated on real human blood samples. A healthy volunteer and a high UA patient provided blood samples with their own consent. The samples were diluted 30 times with 0.1 M phosphate buffer solution of pH 7.0 and used directly during the setup of three electrode cells for electrochemical quantification of UA. UA non-enzymatic sensor performance confirms its potential applicability and reliability for the quantification of UA from biological fluids, and even food products with high probability, as shown in Table 2. A percent relative standard deviation RSD (%) was calculated from the sum of (standard deviation/mean of quantified UA concentration data using three repeated experiments for UA detection) × 100%.

## 4. Conclusions

An easy and rapid method has been developed for quantifying UA in human blood and urine samples by using surface-modified NiCo_2_O_4_ nanostructures. It has been combined with phytochemicals derived from radish white peel. It was determined that a volume of 5 mL of radish white peel extract was optimal for the large-scale synthesis of NiCo_2_O_4_ nanostructures. The CV exhibited a linear range from 0.1 mM to 8 mM, the LSV showed 0.1 mM to 7 mM, and the amperometric signal for the UA sensor showed a linear range from 0.1 mM to 4 mM. We found that the LOD and LOQ of the present non-enzymatic UA sensor were 0.005 mM and 0.008 mM, respectively. Furthermore, the present UA sensor was evaluated in terms of its selectivity, stability, repeatability, and sensitivity. Consequently, a wide linear range and a low limit of detection were achieved. Several factors contribute to the sensor’s performance, including large surface vacancies, abundant chemical states of Co(III), Ni(II), tailored surface morphology, fast charge-transfer rate, and outstanding crystal quality. It has been demonstrated that there is a satisfactory level of detection of UA in human blood samples (healthy and UA patients).

## Figures and Tables

**Table 1:** Performance comparison of the enzyme-free sensor based on NiCo_2_O_4_ nanostructures grown with 5 mL radish (*Raphanus sativus*) extract versus several non-enzymatic UA sensors in the literature.

Sensing Electrode Material	Linear Range (ụM)	Detection of Limit (ụM)	References
PCN ^a^/MWCNT ^b^	0.2–20	0.139	[50]
B-MWCNTS ^c^	62–250	0.65	[51]
Pd/RGO ^d^	6–469.5	1.6	[18]
Au/RGO ^d^	8.8–53	1.8	[52]
PtNi@MoS_2_^e^	0.5–600	0.1	[53]
Cysteic acid	1.0–19	0.36	[54]
Co_3_O_4_	500–3500	100	[56]
Fe_2_O_3_ @Au	100–10,000	0.087	[57]
NiCo_2_O_4_ (30%)/Nano-ZSM-5	0.9–1000	0.7	[58]
AuNPs@GO/PPy/CFP	2–360	1.68	[59]
MWNTs/MGF	300–1000	0.93	[60]
NiCo_2_O_4_ NPs	100–8000	0.005	This work

^a^ Porous g-C_3_N_4_. ^b^ Multi-walled carbon nanotubes. ^c^ Boron-dopped multi-walled carbon naotubes. ^d^ Reduced graphene oxide, ^e^ PtNi bimetallic nanoparticles-loaded nanosheets, Gold nanorod-decorated graphene oxide glassy carbon electrode

**Table 2:** Real sample analysis of UA from various blood samples (Blood 1 = healthy blood sample; Blood 2 = high-level UA patient).

Sample	Added (mM)	Found (mM)	%Recovery	% RSD
Blood 1	-	0.1	-	
-	0.5	0.612 ± 0.002	101.66	0.52
-	1	1.123 ± 0.0023	101.81	0.53
-	2	2.084 ± 0.0017	99	0.49
-	2.5	2.621 ± 0.0013	100.77	0.54
Blood 2	-	1.00	-	-
-	1.5	2.533 ± 0.0022	101.2	0.48
-	2	3.082 ± 0.0019	102.66	0.56
-	2.5	3.514 ± 0.0024	100.28	0.50
-	3	4.063 ± 0.0018	101.5	0.53

## Data Availability

The authors declare that the data supporting the findings of this study are available within the paper.
